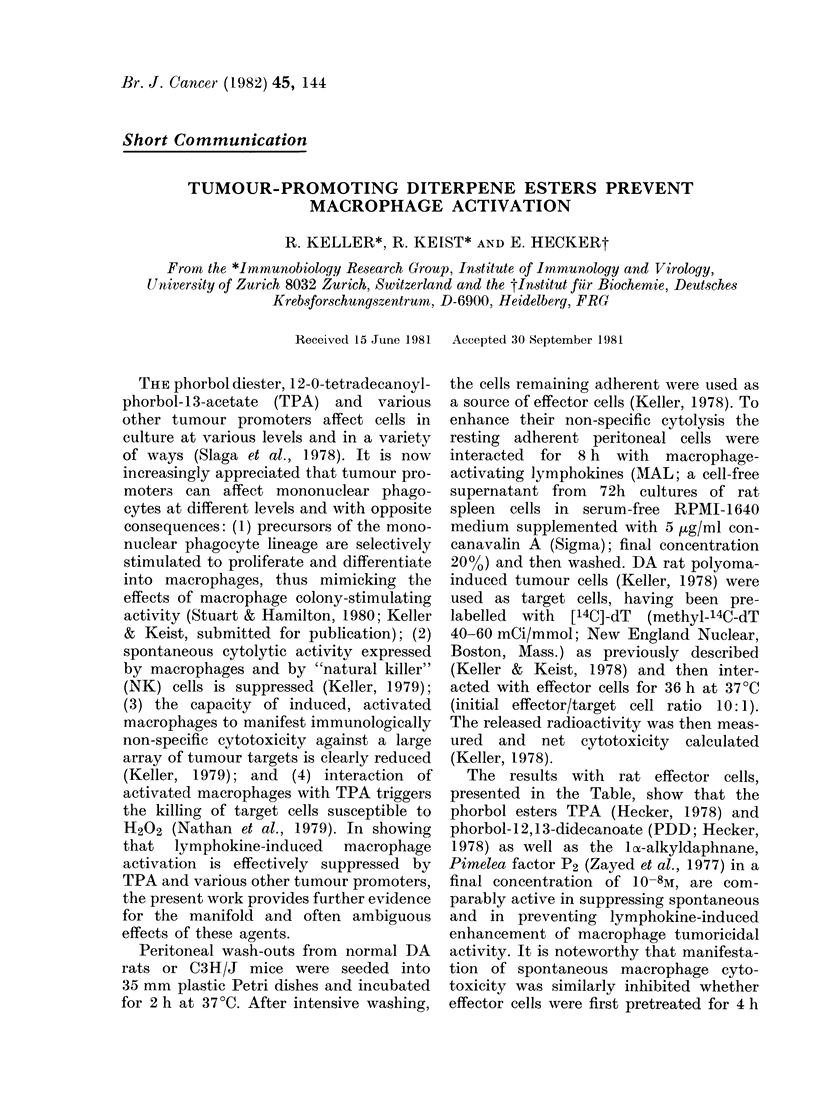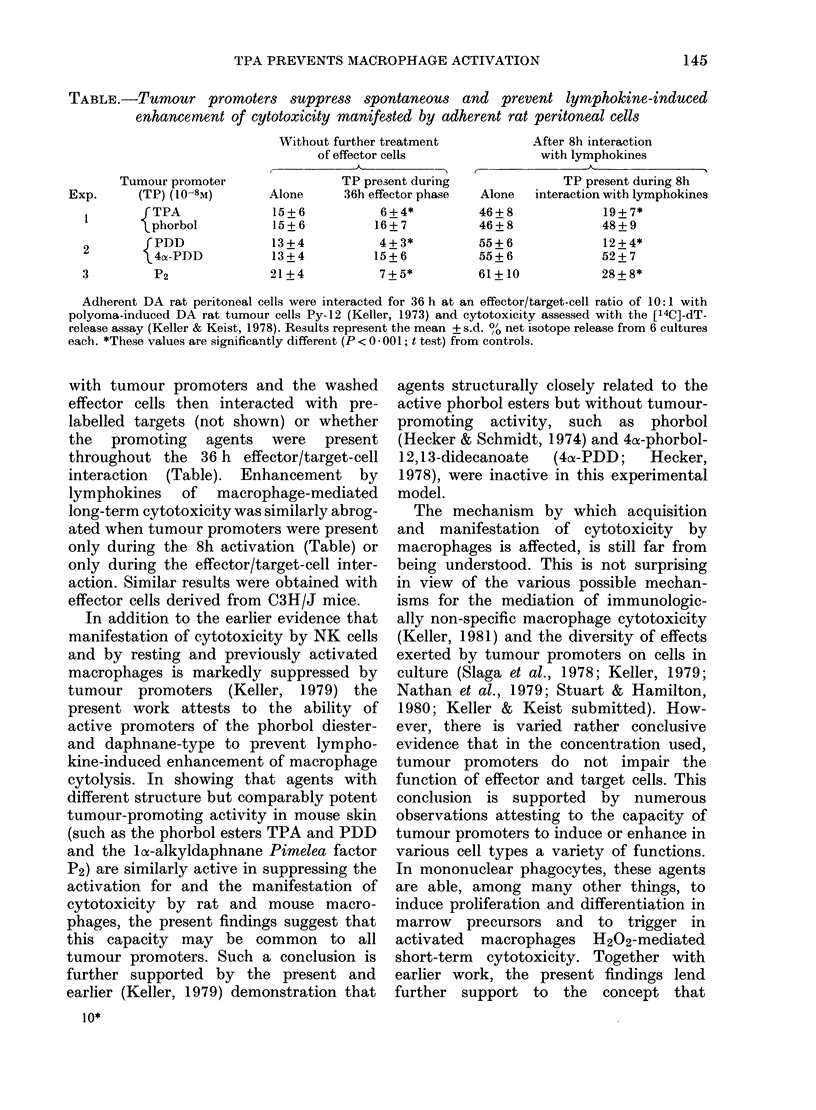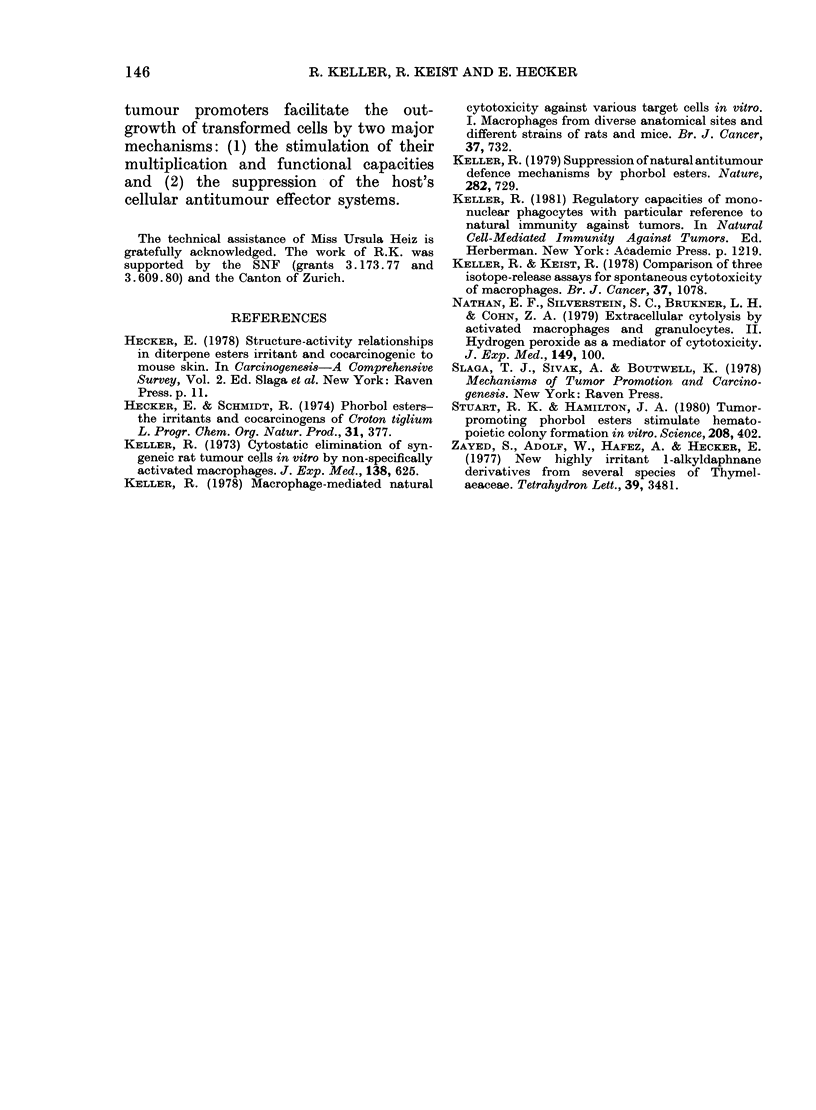# Tumour-promoting diterpene esters prevent macrophage activation.

**DOI:** 10.1038/bjc.1982.18

**Published:** 1982-01

**Authors:** R. Keller, R. Keist, E. Hecker


*Br. J. Cancer* (1982) **45**, 144

***Short Communication***

### TUMOUR-PROMOTING DITERPENE ESTERS PREVENT MACROPHAGE ACTIVATION

R. KELLER*, R. KEIST* AND E. HECKER†

*From the *Immunobiology Research Group, Institute of Immunology and Virology, University of Zurich* 8032 *Zurich, Switzerland and the †Institut für Biochemie, Deutsches Krebsforschungszentrum, D*-6900, *Heidelberg, FRG*

Received 15 June 1981 Accepted 30 September 1981

THE phorbol diester, 12-0-tetradecanoyl-phorbol-13-acetate (TPA) and various other tumour promoters affect cells in culture at various levels and in a variety of ways (Slaga *et al.*, 1978). It is now increasingly appreciated that tumour promoters can affect mononuclear phagocytes at different levels and with opposite consequences: (1) precursors of the mononuclear phagocyte lineage are selectively stimulated to proliferate and differentiate into macrophages, thus mimicking the effects of macrophage colony-stimulating activity (Stuart & Hamilton, 1980; Keller & Keist, submitted for publication); (2) spontaneous cytolytic activity expressed by macrophages and by "natural killer" (NK) cells is suppressed (Keller, 1979); (3) the capacity of induced, activated macrophages to manifest immunologically non-specific cytotoxicity against a large array of tumour targets is clearly reduced (Keller, 1979); and (4) interaction of activated macrophages with TPA triggers the killing of target cells susceptible to $H_2O_2$ (Nathan *et al.*, 1979). In showing that lymphokine-induced macrophage activation is effectively suppressed by TPA and various other tumour promoters, the present work provides further evidence for the manifold and often ambiguous effects of these agents.

Peritoneal wash-outs from normal DA rats or C3H/J mice were seeded into 35 mm plastic Petri dishes and incubated for 2 h at 37°C. After intensive washing, the cells remaining adherent were used as a source of effector cells (Keller, 1978). To enhance their non-specific cytolysis the resting adherent peritoneal cells were interacted for 8 h with macrophage-activating lymphokines (MAL; a cell-free supernatant from 72h cultures of rat spleen cells in serum-free RPMI-1640 medium supplemented with 5 $\mu$g/ml concanavalin A (Sigma); final concentration 20%) and then washed. DA rat polyoma-induced tumour cells (Keller, 1978) were used as target cells, having been pre-labelled with [$^{14}$C]-dT (methyl-$^{14}$C-dT 40–60 mCi/mmol; New England Nuclear, Boston, Mass.) as previously described (Keller & Keist, 1978) and then interacted with effector cells for 36 h at 37°C (initial effector/target cell ratio 10:1). The released radioactivity was then measured and net cytotoxicity calculated (Keller, 1978).

The results with rat effector cells, presented in the Table, show that the phorbol esters TPA (Hecker, 1978) and phorbol-12,13-didecanoate (PDD; Hecker, 1978) as well as the 1$\alpha$-alkyldaphnane, *Pimelea* factor $P_2$ (Zayed *et al.*, 1977) in a final concentration of $10^{-8}$M, are comparably active in suppressing spontaneous and in preventing lymphokine-induced enhancement of macrophage tumoricidal activity. It is noteworthy that manifestation of spontaneous macrophage cytotoxicity was similarly inhibited whether effector cells were first pretreated for 4 h

TABLE.—*Tumour promoters suppress spontaneous and prevent lymphokine-induced enhancement of cytotoxicity manifested by adherent rat peritoneal cells*

| Exp. | Tumour promoter (TP) ($10^{-8}$M) | Without further treatment of effector cells: Alone | Without further treatment of effector cells: TP present during 36h effector phase | After 8h interaction with lymphokines: Alone | After 8h interaction with lymphokines: TP present during 8h interaction with lymphokines |
|---|---|---|---|---|---|
| 1 | TPA | $15 \pm 6$ | $6 \pm 4$* | $46 \pm 8$ | $19 \pm 7$* |
| | phorbol | $15 \pm 6$ | $16 \pm 7$ | $46 \pm 8$ | $48 \pm 9$ |
| 2 | PDD | $13 \pm 4$ | $4 \pm 3$* | $55 \pm 6$ | $12 \pm 4$* |
| | 4α-PDD | $13 \pm 4$ | $15 \pm 6$ | $55 \pm 6$ | $52 \pm 7$ |
| 3 | $P_2$ | $21 \pm 4$ | $7 \pm 5$* | $61 \pm 10$ | $28 \pm 8$* |

Adherent DA rat peritoneal cells were interacted for 36 h at an effector/target-cell ratio of 10:1 with polyoma-induced DA rat tumour cells Py-12 (Keller, 1973) and cytotoxicity assessed with the [$^{14}$C]-dT-release assay (Keller & Keist, 1978). Results represent the mean ± s.d. % net isotope release from 6 cultures each. *These values are significantly different ($P < 0 \cdot 001$; $t$ test) from controls.

with tumour promoters and the washed effector cells then interacted with pre-labelled targets (not shown) or whether the promoting agents were present throughout the 36 h effector/target-cell interaction (Table). Enhancement by lymphokines of macrophage-mediated long-term cytotoxicity was similarly abrogated when tumour promoters were present only during the 8h activation (Table) or only during the effector/target-cell interaction. Similar results were obtained with effector cells derived from C3H/J mice.

In addition to the earlier evidence that manifestation of cytotoxicity by NK cells and by resting and previously activated macrophages is markedly suppressed by tumour promoters (Keller, 1979) the present work attests to the ability of active promoters of the phorbol diester- and daphnane-type to prevent lymphokine-induced enhancement of macrophage cytolysis. In showing that agents with different structure but comparably potent tumour-promoting activity in mouse skin (such as the phorbol esters TPA and PDD and the 1α-alkyldaphnane *Pimelea* factor $P_2$) are similarly active in suppressing the activation for and the manifestation of cytotoxicity by rat and mouse macrophages, the present findings suggest that this capacity may be common to all tumour promoters. Such a conclusion is further supported by the present and earlier (Keller, 1979) demonstration that agents structurally closely related to the active phorbol esters but without tumour-promoting activity, such as phorbol (Hecker & Schmidt, 1974) and 4α-phorbol-12,13-didecanoate (4α-PDD; Hecker, 1978), were inactive in this experimental model.

The mechanism by which acquisition and manifestation of cytotoxicity by macrophages is affected, is still far from being understood. This is not surprising in view of the various possible mechanisms for the mediation of immunologically non-specific macrophage cytotoxicity (Keller, 1981) and the diversity of effects exerted by tumour promoters on cells in culture (Slaga *et al.*, 1978; Keller, 1979; Nathan *et al.*, 1979; Stuart & Hamilton, 1980; Keller & Keist submitted). However, there is varied rather conclusive evidence that in the concentration used, tumour promoters do not impair the function of effector and target cells. This conclusion is supported by numerous observations attesting to the capacity of tumour promoters to induce or enhance in various cell types a variety of functions. In mononuclear phagocytes, these agents are able, among many other things, to induce proliferation and differentiation in marrow precursors and to trigger in activated macrophages $H_2O_2$-mediated short-term cytotoxicity. Together with earlier work, the present findings lend further support to the concept that tumour promoters facilitate the outgrowth of transformed cells by two major mechanisms: (1) the stimulation of their multiplication and functional capacities and (2) the suppression of the host's cellular antitumour effector systems.

The technical assistance of Miss Ursula Heiz is gratefully acknowledged. The work of R.K. was supported by the SNF (grants 3.173.77 and 3.609.80) and the Canton of Zurich.

### REFERENCES

Hecker, E. (1978) Structure-activity relationships in diterpene esters irritant and cocarcinogenic to mouse skin. In *Carcinogenesis—A Comprehensive Survey*, Vol. 2. Ed. Slaga *et al.* New York: Raven Press. p. 11.

Hecker, E. & Schmidt, R. (1974) Phorbol esters–the irritants and cocarcinogens of *Croton tiglium L. Progr. Chem. Org. Natur. Prod.*, **31**, 377.

Keller, R. (1973) Cytostatic elimination of syngeneic rat tumour cells *in vitro* by non-specifically activated macrophages. *J. Exp. Med.*, **138**, 625.

Keller, R. (1978) Macrophage-mediated natural cytotoxicity against various target cells *in vitro*. I. Macrophages from diverse anatomical sites and different strains of rats and mice. *Br. J. Cancer*, **37**, 732.

Keller, R. (1979) Suppression of natural antitumour defence mechanisms by phorbol esters. *Nature*, **282**, 729.

Keller, R. (1981) Regulatory capacities of mononuclear phagocytes with particular reference to natural immunity against tumors. In *Natural Cell-Mediated Immunity Against Tumors*. Ed. Herberman. New York: Academic Press. p. 1219.

Keller, R. & Keist, R. (1978) Comparison of three isotope-release assays for spontaneous cytotoxicity of macrophages. *Br. J. Cancer*, **37**, 1078.

Nathan, E. F., Silverstein, S. C., Brukner, L. H. & Cohn, Z. A. (1979) Extracellular cytolysis by activated macrophages and granulocytes. II. Hydrogen peroxide as a mediator of cytotoxicity. *J. Exp. Med.*, **149**, 100.

Slaga, T. J., Sivak, A. & Boutwell, K. (1978) *Mechanisms of Tumor Promotion and Carcinogenesis*. New York: Raven Press.

Stuart, R. K. & Hamilton, J. A. (1980) Tumor-promoting phorbol esters stimulate hematopoietic colony formation *in vitro*. *Science*, **208**, 402.

Zayed, S., Adolf, W., Hafez, A. & Hecker, E. (1977) New highly irritant 1-alkyldaphnane derivatives from several species of Thymelaeaceae. *Tetrahydron Lett.*, **39**, 3481.